# Polymer/Iron Oxide Nanoparticle Composites—A Straight Forward and Scalable Synthesis Approach

**DOI:** 10.3390/ijms160819752

**Published:** 2015-08-20

**Authors:** Jens Sommertune, Abhilash Sugunan, Anwar Ahniyaz, Rebecca Stjernberg Bejhed, Anna Sarwe, Christer Johansson, Christoph Balceris, Frank Ludwig, Oliver Posth, Andrea Fornara

**Affiliations:** 1 SP, Technical Research Institute of Sweden, Box 5607, SE-114 86 Stockholm, Sweden; E-Mails: jens.sommertune@sp.se (J.S.); abhilash.sugunan@sp.se (A.S.); anwar.ahniyaz@sp.se (A.A.); 2 Department of Engineering Sciences, Solid State Physics, Uppsala University, SE-751 21 Uppsala, Sweden; E-Mail: Rebecca.Bejhed@angstrom.uu.se; 3 Acreo Swedish ICT AB, Box 53071, SE-400 14 Göteborg, Sweden; E-Mails: Anna.Sarwe@acreo.se (A.S.); Christer.Johansson@acreo.se (C.J.); 4 Institute of Electrical Measurement and Fundamental Electrical Engineering, TU Braunschweig, D-38106 Braunschweig, Germany; E-Mails: c.balceris@tu-bs.de (C.B.); f.ludwig@tu-bs.de (F.L.); 5 Physikalisch-Technische Bundesanstalt, 10587 Berlin, Germany; E-Mail: oliver.posth@ptb.de

**Keywords:** iron oxide nanoparticle, multi core, single core, nanocomposite, polymer encapsulation

## Abstract

Magnetic nanoparticle systems can be divided into single-core nanoparticles (with only one magnetic core per particle) and magnetic multi-core nanoparticles (with several magnetic cores per particle). Here, we report multi-core nanoparticle synthesis based on a controlled precipitation process within a well-defined oil in water emulsion to trap the superparamagnetic iron oxide nanoparticles (SPION) in a range of polymer matrices of choice, such as poly(styrene), poly(lactid acid), poly(methyl methacrylate), and poly(caprolactone). Multi-core particles were obtained within the Z-average size range of 130 to 340 nm. With the aim to combine the fast room temperature magnetic relaxation of small individual cores with high magnetization of the ensemble of SPIONs, we used small (<10 nm) core nanoparticles. The performed synthesis is highly flexible with respect to the choice of polymer and SPION loading and gives rise to multi-core particles with interesting magnetic properties and magnetic resonance imaging (MRI) contrast efficacy.

## 1. Introduction

In the last few decades there has been a tremendous development in the synthesis and use of magnetic iron oxide nanoparticles, mainly due to their interesting magnetic properties at the nanoscale and relative low toxicity [1]. The synthesis routes to obtain small iron oxide nanoparticles with superparamagnetic behavior at room temperature (SPION) can be either water-based or organic solvent-based [2]. Most applications require a specific surface modification of the bare SPION in order to obtain a stable colloidal suspension in aqueous media [3] or to incorporate them into polymeric structures [4]. Magnetic nanoparticles are today under investigation for a variety of applications, mainly in the biomedical area [5], ranging from detection of biomolecules [6] to drug delivery [7], from Magnetic Resonance Imaging [8] to protein purification [9] or a combination of them [10].

In this paper we will use the commonly accepted term “single-core” to describe an individual nanoparticle, and “multi-core” to describe a collection of cores held by a matrix forming a fixed structure [11]. Single-core and multi-core magnetic particles may exhibit very different magnetic properties due to their average core size [12] or to magnetic interaction between the magnetic cores [11]. Besides basic magnetic properties, single- and multi-core magnetic nanoparticles may exhibit very different MRI contrast properties that are very dependent on the surface modification and coating materials [13]. Multi-core SPIONs are used as contrast agents for T2 weighted MR imaging, wherein the magnetic particles suppress the transverse proton relaxation signals from the surrounding tissue. Potential of SPIONs as contrast agents for MRI can be determined from the longitudinal (R1) and transverse (R2) proton relaxivity measurements. High values of R2 and R2/R1 ratio are desired.

When multi-core nanoparticles are desired, a common approach is to use a polymeric matrix to entrap several magnetic cores. Synthesis methods for polymer/SPION hybrid multicore particles, where the polymer is not only intended as a stabilizer for SPION, have been reported in literature and can be divided in to three major approaches: (a) the incorporation of SPIONs into a forming polymer phase, e.g., polymerization in the presence of the nanoparticles; (b) SPION formation from iron salts in an existing polymer particle and (c) the trapping within a precipitating polymer in the so-called emulsion-solvent-evaporation process (ESE). All of these processes have their advantages and disadvantages [14].

The ESE process for magnetic particles was first reported by Tanyolaç and Özdural [15] who produced hybrid beads in the size range of 125–250 µm. Hamoudeh *et al*. [16] reported a modified process, yielding magnetite/poly(lactic acid) hybrids in the size range between 320 nm and 1.5 µm, based on earlier poly(caprolactone) hybrids between 3 and 23 µm [17]. Lee *et al*. [18] reported 90–180 nm hybrid particles based on a solvent diffusion process rather than a solvent evaporation process. The major advantages of the ESE process are the wide choice of polymers. Here, pre-synthesized polymers can be used, even such that cannot be synthesized in an aqueous environment such as poly(lactic acid) (PLA) and poly(caprolactone) (PCL).

In this paper we present the preparation of multi-core magnetic hybrid particles based on the ESE method in order to investigate the structure/magnetic properties relationship and their potential contrast enhancement in MRI, based on their NMR relaxivities.

## 2. Results and Discussion

### 2.1. Preparation of Single-Core SPION and Multi-Core Nanocomposite Spheres

**Figure 1:**
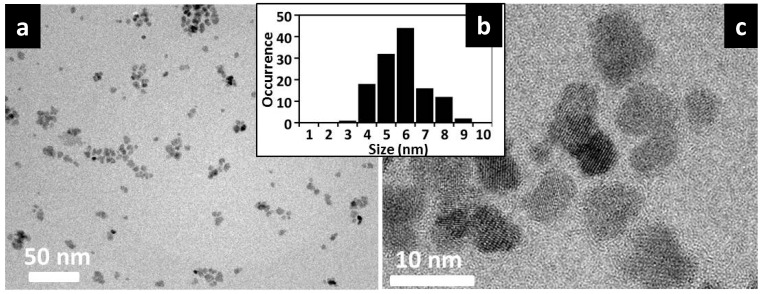
(**a**) Representative TEM (Transmission electron microscope) image of the SPION cores; (**b**) Size histogram generated from particle size analyses from several TEM images; (**c**) High-resolution TEM image showing well developed lattice fringes.

Transmission electron microscope (TEM) images show that the SPIONs are well crystallized and have a small size. Image analysis from multiple TEM images show that the nanoparticles have mean size of about 6 nm (Figure 1). High-resolution TEM show well developed lattice fringes, showing good crystallinity. The small size is indicative of superparamagnetic behavior. Nevertheless, there is empirical evidence that small crystal size leads to low magnetic moments in SPIONs due to dominance of surface effects [19]. However, increasing the crystal size to increase their magnetic moments leads to dominance of Brownian relaxation over Néel relaxation; in other words loss of super-paramagnetic behavior.

**Figure 2:**
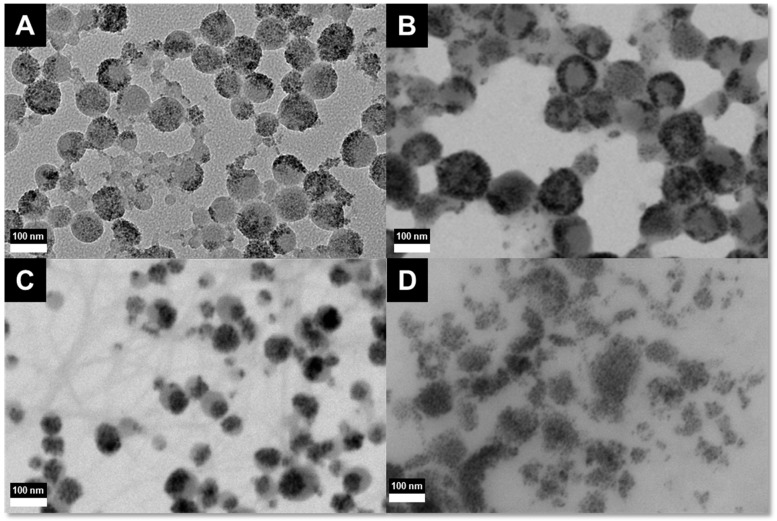
TEM micrograph of sample **A**, STEM (scanning transmission electron microscope) micrographs of sample **B**, **C**, and **D** (scale bars 100 nm).

One of the ways to overcome this dilemma is to synthesize and utilize multi-core particles, where the individual magnetic cores do not show significant magnetic interactions. With this aim we produced composite nanoparticles consisting of multiple magnetic cores within a larger polymeric nanoparticle. SPIONS were trapped into different polymers and an overview of the resulting samples to be discussed is shown in Table 1. Figure 2 shows the TEM micrograph of sample A and the STEM micrographs of samples B, C, and D. The micrographs of sample A reveal a somewhat inhomogeneous distribution of the SPION in the poly(styrene) (PS) matrix. However, there are neither “empty” PS-spheres nor free SPION observed.

**Table 1:** Overview of produced multicore samples.

Sample Code	Core	Polymer Matrix
A	Oleic acid stabilized iron oxide	poly(styrene)
B	Oleic acid stabilized iron oxide	poly(lactic acid)
C	Oleic acid stabilized iron oxide	poly(methyl methacrylate)
D	Oleic acid stabilized iron oxide	poly(caprolactone)

The same preparation method was applied to produce PLA hybrid spheres (sample B), PMMA hybrid spheres (sample C), and PCL spheres (sample D). Due to the lower glass transition temperature (T_g_) of PCL, and PLA, the particles are harder to image and melt during imaging. Their imaging in the STEM shows spherical and “loaded” polymer particles, that are somewhat fused due to sample preparation. For sample D however, hardly any polymer could be observed. Still, the images allow the assumption, that even these particles have a spherical shape in aqueous dispersion.

**Figure 3:**
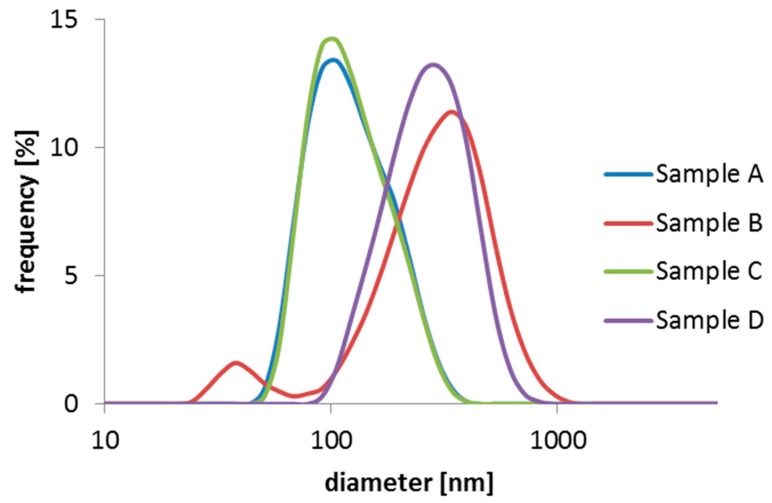
DLS size distribution by intensity of samples A, B, C, and D.

**Figure 4:**
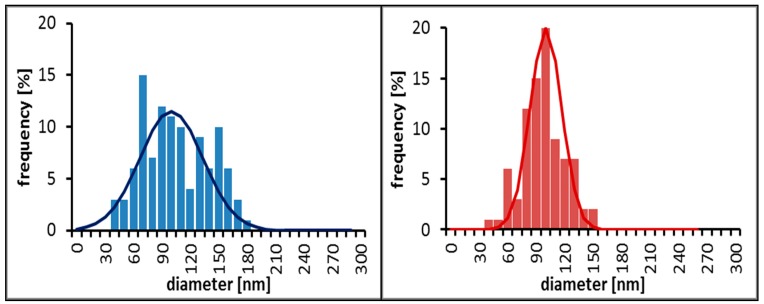
Size distribution based on TEM and STEM micrographs of Sample A (**left**) and Sample B (**right**).

The size distribution of all samples was also investigated by means of Dynamic Light Scattering (DLS) in aqueous dispersion. Figure 3 shows the DLS results of Sample A, B, C, and D. Unlike the TEM images and subsequent size analysis statistics in the dry-state (Figure 4), the DLS measurement reveals the “water-swollen” size.

A comparison of the DLS and TEM sizes show that the measured difference in the DLS Z-average (131 nm for Sample A and 237 nm for Sample B) is not observed by TEM (both samples average at 100 nm). This most likely could be attributed to swollen *versus* dry size of the particles, although an overestimation in DLS size due to intensity weighted statistics being dominated by a few large particles cannot be ruled out, since the obtained size distributions from the TEM images are number weighted.

**Figure 5:**
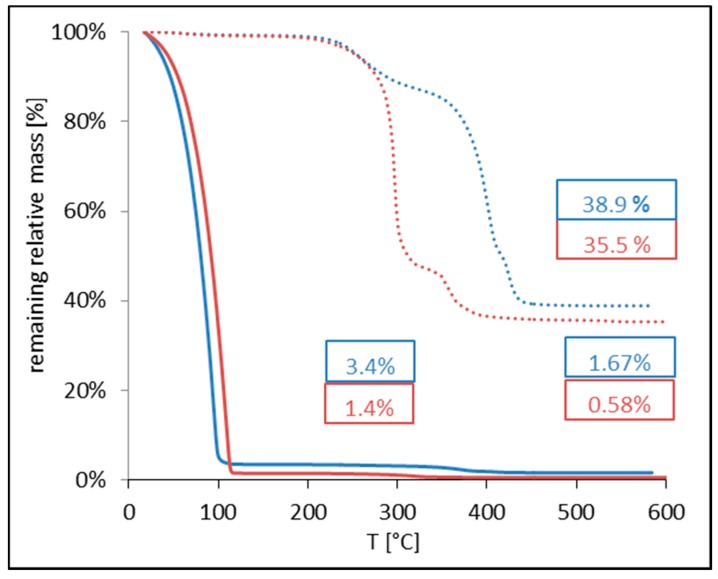
TGA curves of aqueous dispersions (full line) and dried particles (dotted line) of Sample A (blue) and Sample B (red).

The thermal decomposition (see Figure 5) shows that Sample A and B show different amounts of polymer, stabilizers, and iron oxide in the dispersions. Whereas Sample A contains about 96.6 wt % water, Sample B contains ~98.6 wt % water. This can be explained by varying efficiency of the magnetic collection. However, looking at the dried samples (dotted lines in Figure 5), the analysis shows that Sample A consists of ~15 wt % stabilizer, ~45 wt % PS, and ~39 wt % SPION. Sample B on the other hand has a somewhat higher polymer amount (~53 wt %), ~12 wt % stabilizer and ~35 wt % SPION. The TGA curves clearly show the lower heat stability of PLA as compared to PS.

We judge this entrapment process to be easily scalable and highly versatile. By preparing higher volumes of emulsions with SPIONs in the oil phase, larger amounts of hybrid spheres can be produced. Further, the choice of the polymer is vast and only limited by the solubility in a hydrophobic solvent with a higher vapor pressure as water. Even a “switched system” with hydrophilic SPIONs in a water-soluble polymer via a water in oil emulsion seems possible.

### 2.2. Magnetic Characterization of Multicore Nanocomposite Spheres

In order to test our main hypothesis of multicore SPION particles retaining their fast magnetic relaxation, Sample A and B were analyzed in more detail with respect to their magnetic behavior. Here, sample A (PS) and B (PLA) were chosen due to the fact that PS is a typical polymer used in commercial particles of this kind and PLA was chosen since it is a biodegradable polymer, allowing for *in vivo* degradation in potential future applications. Furthermore Sample A is a typical hydrophobic polymer and Sample B is a typical hydrophilic polymer, which is relevant because the polymer beads could show different swelling and other physicochemical behavior in aqueous suspension. TEM analysis revealed that the average iron oxide core nanocrystal size of Sample A was 8.7 and 9.4 nm for Sample B (data not shown).

**Figure 6:**
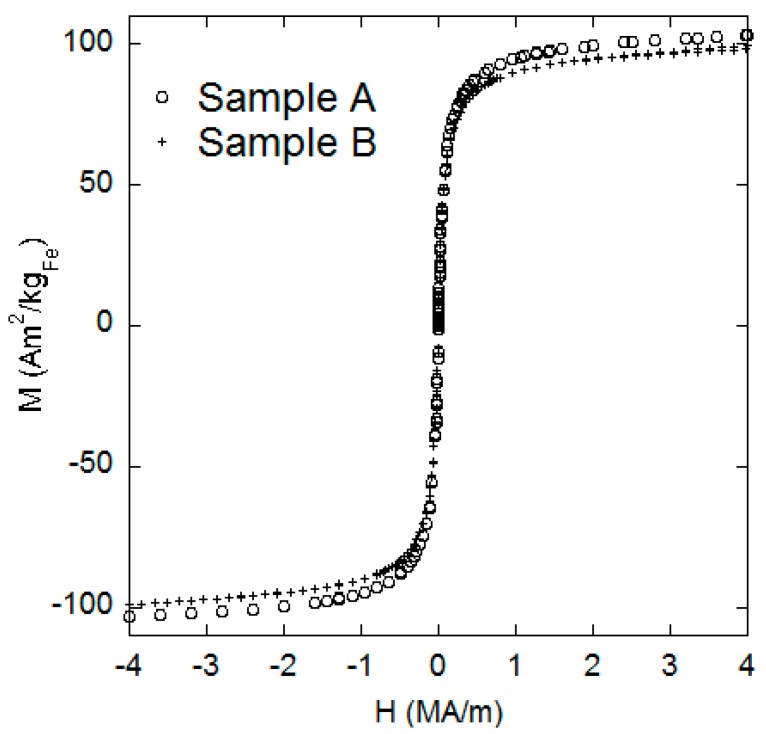
DC magnetization *versus* applied field for samples A and B.

Firstly, we tested the DC-magnetization *versus* field of Samples A and B at 300 K (see Figure 6). We could not observe any hysteresis in both the samples. The intrinsic saturation magnetization is 103 Am^2^/kg_Fe_ for Sample A and 98 Am^2^/kg_Fe_ for Sample B. This corresponds to 75 and 71 Am^2^/kg respectively for the iron oxide nanoparticles, assuming magnetite phase. The small variation in intrinsic saturation magnetization is probably due to batch to batch variation during core synthesis. Background magnetic moments due to the sample cup and water content have been removed.

**Figure 7:**
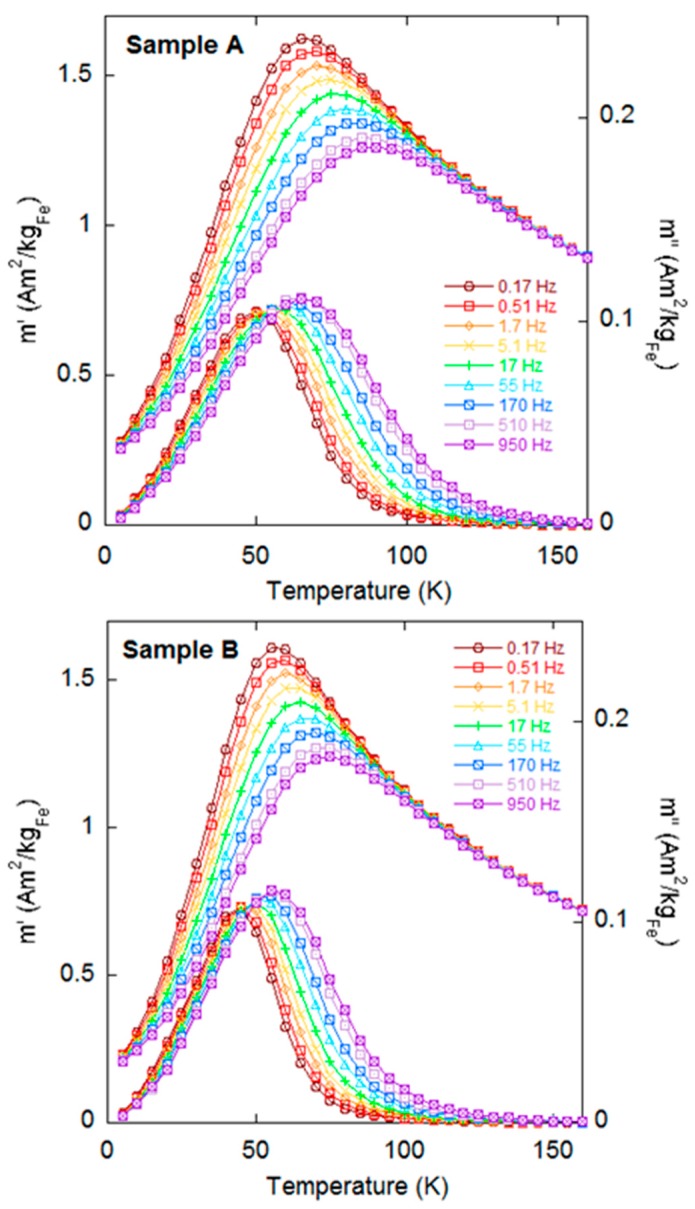
AC magnetization *versus* temperature for samples A and B. The different curves correspond to different frequencies of the AC magnetic field. The AC magnetic field amplitude is 320 A/m.

We also measured low field AC magnetization *versus* temperature at different frequencies of the applied AC magnetic field. In such measurements, a low amplitude AC magnetic field is applied and the dynamic magnetic response from the sample is measured as a function of temperature. In Figure 7a,b, the in- and out-of-phase AC magnetization is plotted *versus* temperature for Sample A and Sample B, respectively, the different curves correspond to different AC magnetic field frequencies. The superparamagnetic behaviour remains for both samples until low temperature and blocking/freezing of nanocrystal magnetic moments is not observed until the temperature has reached below about 150 K.

The sample blocking/freezing temperature, *T_f_*, can be extracted from the out-of-phase AC magnetization, *m’’*, curves as the temperature corresponding to half the maximal *m’’* value (towards higher temperatures), *i.e*., one *T_f_* for each frequency. By plotting ln(1/2π*f*) *versus* 1/*T_f_*, from the ACS measurements, and fitting it to an Arrhenius equation shown below (assuming non-interacting particles), information regarding the magnetic anisotropy constants *K* and possible interparticle interactions can be obtained.

(1) $$\text{τ}\left(T_{f}\right)={\text{τ}}_{0}exp\left(KV_{cp}/{\text{κ}}_{B}T_{f}\right)$$

Here, τ_0_ is a microscopic relaxation time expected to be in the order of 10^−12^–10^−11^ s, *V_cp_* is the mean volume of the nanocrystals and *k_B_* is Boltzmann constant.

**Figure 8:**
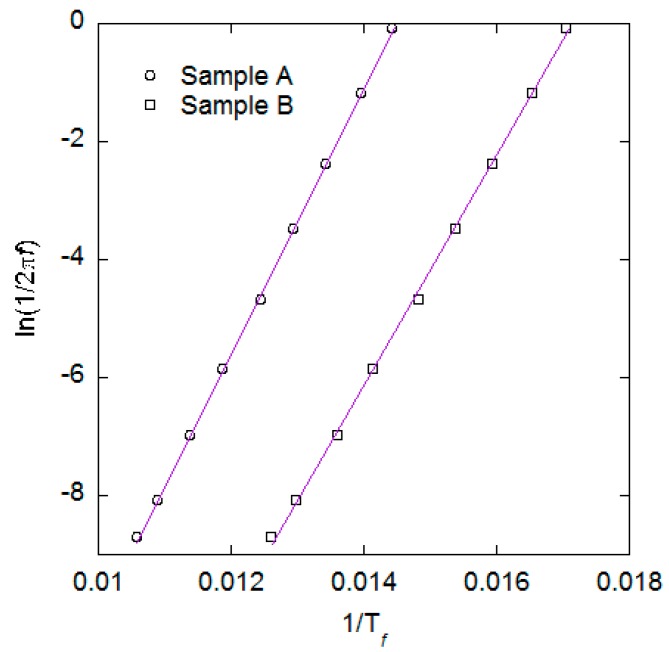
Data extracted from the ACS *versus* temperature measurements for samples A and B. Data have been fitted using straight lines.

Figure 8 shows data extracted from the ACS *versus* temperature measurements. The data points have been fitted to a straight line equation yielding τ_0_ in the order of 10^−15^ s for both samples, indicating some interparticle interactions. By using a diameter of 8.7 nm for Sample A and 9.4 nm for Sample B we obtain rather large anisotropy constants of 90 and 62 kJ/m^3^ respectively. These values are higher than bulk values for both magnetite and maghemite, indicating an anisotropy contribution from e.g., the large surface/volume ratio. These “unphysical” values of τ_0_ and *K_cp_* are a clear sign of inter-particle interactions.

In ACS *versus* frequency measurements, a low field amplitude excitation AC magnetic field is applied and the dynamic magnetic response from the sample is measured. The dynamic response is divided into one component that is in-phase with the excitation field (the real part, χ′) and one component that is 90° shifted with respect to the excitation field (the imaginary part or also called the magnetic loss term, χ″). In this study the DynoMag system (1 Hz–250 kHz) was used together with a high frequency (HF) susceptometer (10 kHz–10 MHz) [20,21,22]. These measurements give magnetic relaxation information of the investigated magnetic nanoparticle systems up to 10 MHz. From the ACS relaxation spectra it is possible to extract various pertinent data for the magnetic nanoparticle systems [12,23], for instance, type of magnetic relaxation (Brownian or Néel relaxation) by measuring on both free and immobilized magnetic nanoparticles.

**Figure 9:**
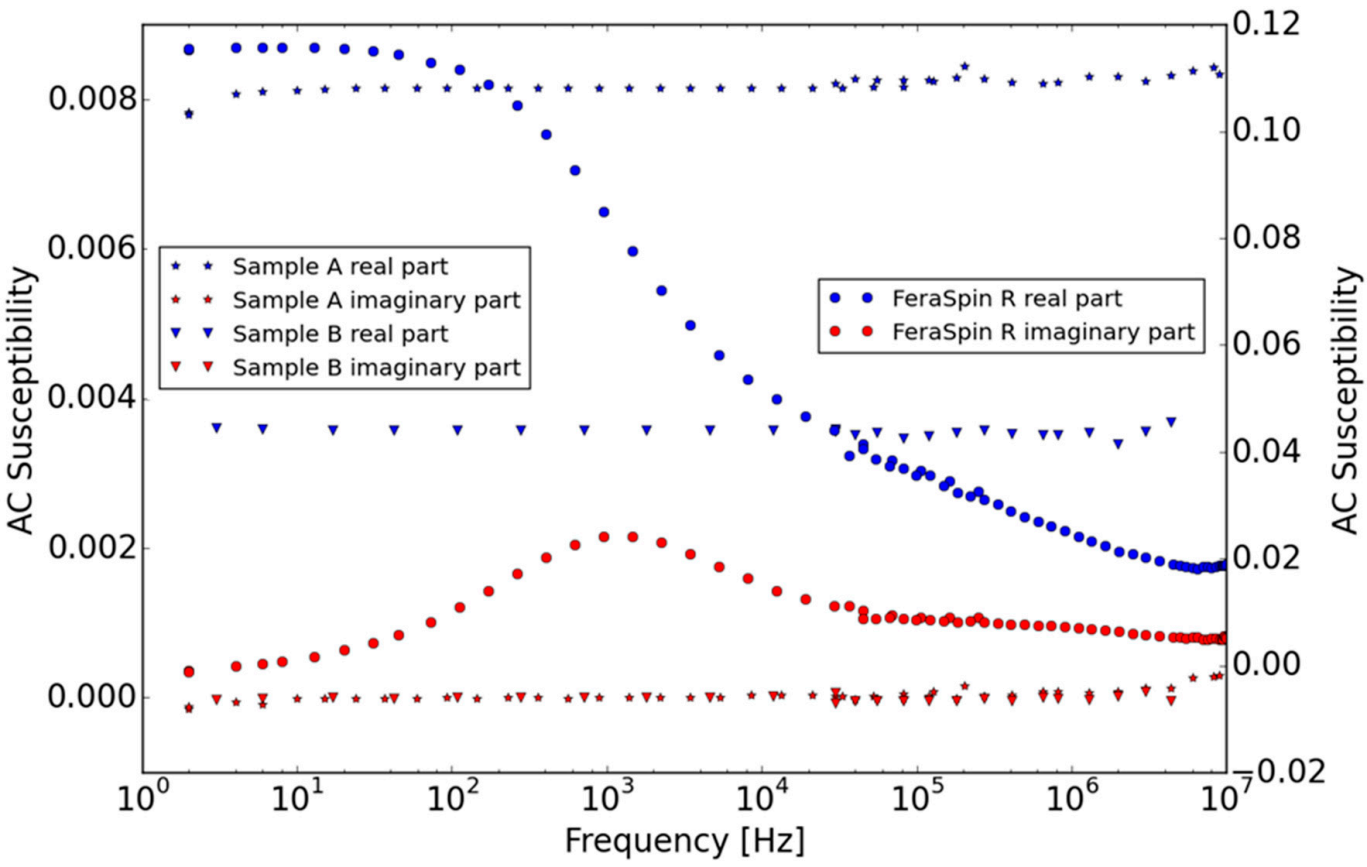
Real and imaginary components of the AC susceptibility *versus* frequency of samples A and B (left y-scale), and FeraSpin™-R (right y-scale) at room temperature. No magnetic relaxation peak is observed for samples A and B. As a comparison, a commercial multi-core sample, FeraSpin™-R from nanoPET (right scale), shows a well-developed relaxation peak, indicating a Brownian relaxation frequency in the range of 1 kHz.

In Figure 9, χ′ and χ″ are shown *versus* frequency at room temperature for Samples A and B and compared to a commercially available multicore sample, FeraSpin™-R (from nanoPET Pharma GmbH, Berlin, Germany). Samples A and B show almost identical response with a constant χ′ and χ′ʹ being close to zero in the whole measured frequency range up to 10 MHz. This is typical of fast Néel relaxation where the excitation frequency is much smaller than the Néel relaxation frequency (=1/(2πτ_N_), where τ_N_ is the Néel relaxation time). From previous measurements on similar iron oxide nanoparticles we conclude that these results are in agreement with the TEM data of nanocrystal sizes in the range of 8–9 nm. The value of χ′, normalized with the iron concentration, are the same for sample A and B, indicating almost the same nanocrystal size distribution and intrinsic saturation magnetization of sample A and B which are consistent with the TEM and magnetization *versus* field analysis. As expected, the ACS *versus* frequency data of the core nanocrystals (data not shown) also did not show any features in the entire range of frequencies.

Thus, both samples A and B exhibit fast Néel relaxation at room temperature (Néel relaxation frequencies above 10 MHz) and encapsulation did not significantly affect the magnetic relaxation. A small increase of χ′ʹ in the range of 2 MHz indicates that the excitation frequency is approaching the Néel relaxation frequency that should be above 10 MHz. This lack of a clear χ′ʹ peak within the measured frequency range can be explained by the nanocrystal sizes in these samples being smaller than the critical nanocrystal size for thermal blocking of the internal magnetization [24]. We estimated based on our experience with such multi-core systems that this critical nanocrystal size is in the range of 18 nm for non-interacting magnetite nanocrystals in a multi-core particle with size of about 100 nm dispersed in water at room temperature, using the concept of effective relaxation of Brownian and Neel relaxations [20,22]. Furthermore, from the electron microscopy results of the samples, it is seen that the nanocrystals in the multi-core particles of Sample A and B are not densely packed. Dense packing of nanocrystals in a multi-core particle system results in high magnetic interactions between the nanocrystals, which increase the Néel relaxation time and can result in thermally blocking of the nanocrystals in multi-core particle structures. This is evident in the dynamic response of FeraSpin™-R showing Brownian relaxation even though the nanocrystals are of similar size. In this case the nanocrystals are densely packed in the multi-core structure and experience high magnetic interactions that increase the Néel relaxation time and the susceptibility when normalized to the iron concentration [12,24]. The Brownian relaxation frequency for this multi-core particle system is in the range of 1 kHz corresponding to a particle size of 70 nm (in good agreement with DLS data for the FeraSpin™-R).

**Figure 10:**
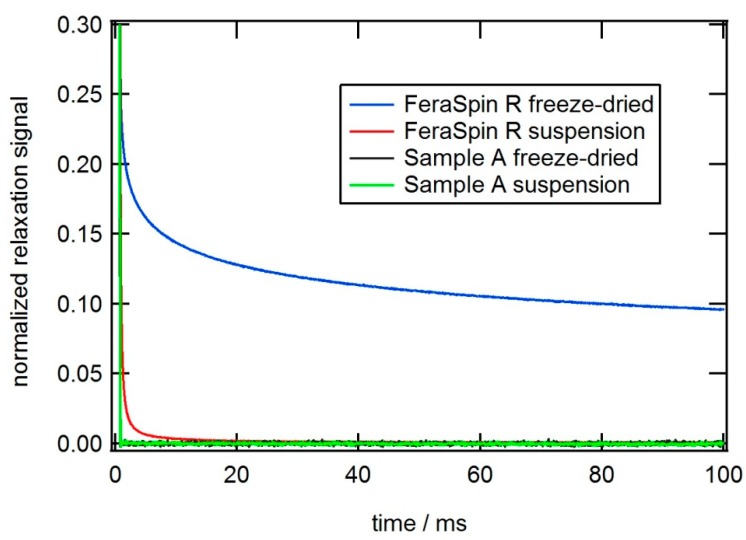
Normalized signal of the relaxation cycle of a MRX (magnetorelaxometry) measurement recorded on sample A in comparison to FeraSpin™-R with the fluxgate setup. No difference between suspended and immobilized particles is discernable for Sample A in contrast to FeraSpin™-R.

We also performed magnetorelaxometry (MRX) to determine the magnetic relaxation mechanism to confirm superparamagnetic behavior in Samples A and B, compared to FeraSpin™-R. In MRX measurement, a magnetic nanoparticle sample is polarized for 1–2 s in a static magnetic field of a few mT [25,26]. After abruptly switching off the field, the particles relax via Néel or Brownian rotation and the decay of the magnetic moment is recorded. Thus, MRX provides information on the relaxation times. For immobilized samples, the magnetic nanoparticles relax via the internal Néel mechanism, whereas for magnetic nanoparticle suspensions both Brownian and Néel relaxation can take place whereby the faster of the two dominates. The MRX measurements on samples A, B and core only, show no analyzable decay of magnetic moment for the fluid samples or the immobilized particles (as shown for Sample A in Figure 10). This indicates that the effective time constant is so short that the magnetic moments can follow the magnetic field changes almost instantaneously. This is in contrast to the behavior of FeraSpin™-R which exhibits distinct relaxation behavior in spite of similar crystallite sizes of 5–7 nm. For FeraSpin™-R, a clear relaxation signal is observed for both the suspended and the freeze-dried reference sample. The slower relaxation for the freeze-dried sample in which only Néel relaxation can take place indicates that the dynamics of at least part of the nanoparticles in FeraSpin™-R suspensions is dominated by Brownian rotation. A possible explanation is that the magnetic cores of samples A and B do not undergo the dipolar coupling that is responsible for the observed relaxation signal in FeraSpin™-R. Reduced dipolar coupling can originate either in larger inter-crystallite distances or in smaller magnetic moments per crystallites. Further investigations will confirm which of these factors contribute to the reduced dipolar coupling in our samples compared to FeraSpin™-R.

Finally we investigated the nuclear magnetic resonance (NMR) relaxivity of these multicore nanoparticles for a preliminary assessment of their magnetic resonance imaging (MRI) contrast enhancement. The obtained relaxivity data are shown in Table 2. It is reported that forming clusters of multiple magnetic crystals show enhanced R2 relaxivities compared to single cores [27]. In order to test this, we also surface modified a batch of the core nanocrystals with poly(acrylic acid) to obtain an aqueous dispersion and compared the NMR relaxivities of the multicore dispersions with single core dispersion as well as with commercial T2 contrasts agents consisting of similar multicore nanoparticle dispersions.

The R2 relaxivities as well as the R2/R1 ratios of the multicore magnetic nanocomposites (Samples A and B) are indeed much higher compared to the values for aqueous suspension of the single core nanocrystals. Also in comparison to commercial MRI contrast agents (Resovist^®^ and FeraSpin™-R), which are established as *in vivo* contrast agents for T2-weighted MR imaging, the R2 relaxivity and R2/R1 ratios of Sample A and B are higher. This is indicative of a higher T2 contrast efficacy of Sample A and Sample B as compared to Resovist^®^ and FeraSpin™-R.

The flexibility in choice of polymer (for e.g., bio- or *in vivo* degradable polymers) combined with good NMR relaxivity performance makes the particles presented in this study potential candidates for the development of new contrast agents for T2 weighted MRI. Further studies involving the detailed investigation of their biocompatibility, *in vivo* behavior and other parameters, have to be undertaken to confirm this potential and to evaluate whether these particles are suitable as MRI contrast agents and could provide an added value to the currently existing agents.

**Table 2:** NMR relaxivities (0.94 T, 39 °C in water).

Sample	R1 (mmol^−1^·s^−1^)	R2 (mmol^−1^·s^−1^)	R2/R1
Core nanocrystals	9.1	61.9	6.8
Sample A	7.0	286.1	41.0
Sample B	4.6	285.9	61.7
FeraSpin™-R	10.4	185.2	17.8
Resovist^®^ [28]	12.3	188	15.3

## 3. Experimental Section

Poly(styrene) (average *M*_W_ 250,000 Da) and poly(methyl methacrylate) (typical *M*_W_ 350,000 Da) were obtained from Sigma Aldrich. Poly(lactic acid) (*M*_W_ 40,000–70,000 Da) and poly(caprolactone) (*M*_W_ 43,000–50,000 Da) were purchased at PolySciences (Eppelheim, Baden-Württemberg, Germany). Poly(styrene-alt-maleic acid) sodium salt solution, was purchased from Sigma Aldrich (Schnelldorf, Bavaria, Germany) and Kemsurf DSA was received by Lankem (Dukinfield, Cheshire, UK). Iron acetylacetonate (97%), sodium borohydride (98%), oleic acid (90%), and poly(acrylic acid) (*M*_W_ = 2000 Da) were purchased from Aldrich (Schnelldorf, Bavaria, Germany).

SPION hybrid particles were produced in a two step procedure. First, iron oxide nanoparticles were produced by a reduction method reported in the literature [29]. In brief, 5.65 g (16 mmol) of iron acetylacetonate was added to 1000 mL of the mixed solvent, as detailed in the literature report. To this solution, 12.1 g (0.32 mol) of sodium borohydride was added quickly and the mixture was stirred overnight at 150 rpm. The black precipitates were collected by centrifugation and rinsed with deionized water. A solution of oleic acid (100 g; 0.35 mol) in chloroform was added to the suspension and ultrasonicated. The nanoparticles migrated to the chloroform phase and were collected by precipitation with ethanol followed by re-dispersion in chloroform.

**Figure 11:**
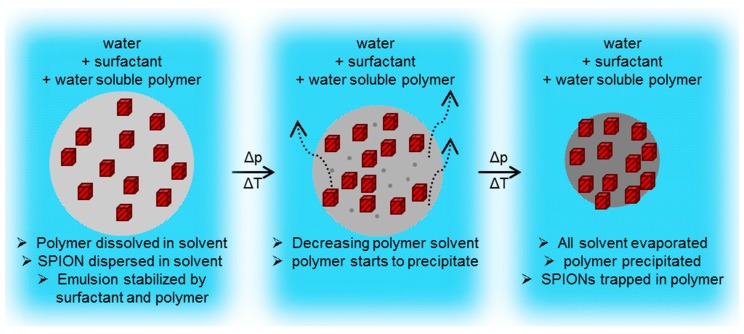
Schematic formation of SPION/polymer hybrid particle, starting from an oil in water emulsion droplet (**left**), proceeding with a high polymer concentration droplet (**middle**) and the formation of a solid polymer sphere with trapped SPIONs (**right**).

Following, the SPION/chloroform dispersion was used as the polymer solvent/oil phase in the emulsion solvent evaporation process (ESE), schematically shown in Figure 11.

Four separate SPION dispersions in chloroform were prepared as described above, and were used to dissolve the desired polymer, namely poly(styrene) (PS), poly(lactic acid) (PLA), poly(methyl methacrylate) (PMMA), or poly(caprolactone) (PCL) to yield a chloroform solution containing 2.5 wt % SPION and 2.5 wt % polymer. Following, the oil phase was transferred to an aqueous solution, containing sodium dodecyl sulfonate (SDS) and poly(styrene-alt maleic acid) sodium salt (0.7 and 0.35 wt % respectively). The mixture was emulsified by means of ultrasonication for 30 min with a Sonics Vibracell at 40% Amplitude in an ice bath. The formed emulsion was transferred to a round-bottom flask and the chloroform was evaporated in a rotary evaporator. The evaporation process was started at 35 °C and 500 mBar, gradually decreasing pressure and increasing temperature to 50 °C and 100 mBar to ensure complete removal of chloroform. The obtained dispersion was cooled to 4 °C in a fridge and subsequently placed on a magnet over night to collect the particles. The next morning, the supernatant was discarded and the collected particles were re-dispersed in deionized water.

In magnetorelaxometry (MRX) measurement, a magnetic nanoparticle (MNP) dispersion is placed in a static magnetic field followed by abrupt switching off of the field and the decay of the magnetic moment is recorded. Thus, MRX provides information on the relaxation times for fluid samples or for immobilized MNPs. The MRX curves can be described by the moment superposition model [30] by which the particle properties, such as the magnetic core size, the hydrodynamic size distribution and the magnetic anisotropy can be determined.

The lower limit of the accessible time constants is given by the switch-off time of the magnetic field, the dead time required to recover the SQUID sensor electronics [31,32] or if fluxgates [33] are used, the bandwidth of the used sensor. The lower limit of a few 100 µs corresponds to a MNP core size of about 17 nm depending on the effective anisotropy constant. Smaller particles cannot be measured by the present MRX setup. The upper limit for our MRX is given by the measurement time which is typically a few seconds.

NMR relaxivity values were determined by measuring the T1- and T2-relaxation times of aqueous nanoparticle suspensions at concentrations of 1.5, 1.0, 0.5, and 0.1 mM Fe using a nuclear magnetic resonance pulse spectrometer (miniSpec mq40; Bruker Biospin; Germany). The measurements were performed at 0.94 T and 39 °C. Each dilution was measured twice. Relaxivity values (R1 and R2) were then calculated by estimating the slope of the linear regression of the relaxation rate 1/*Ti* (*i* = 1, 2) *versus* the concentration.

## 4. Conclusions

The ESE process is a powerful tool for the entrapment of SPIONs into polymer spheres. In contrast to *in situ* polymerization processes, such as mini-emulsion polymerization, this process enables a wider choice of polymers and can produce hybrid particles of polymers that have to be synthesized in a water-free environment. The loading of the hybrid spheres can be regulated easily by balancing the SPION/polymer ratio in the starting dispersion. The emulsion preparation with ultrasonication yields small droplet sizes and accordingly small hybrid particles after the evaporation process. We find that multicore SPION-polymer nanocomposite particles so obtained show extremely fast magnetic relaxation. Furthermore, these dispersions could find potential applications as contrast agents for T2 weighted MR imaging due to their enhanced R2 relaxivity values and high R2/R1 ratios compared to commercially established iron oxide based T2 contrast agents.
